# Biological basis for adoption of an isoline of *Telenomus remus* (Hymenoptera: Scelionidae) for an augmentative biological-control program for *Spodoptera frugiperda* (Lepidoptera: Noctuidae)

**DOI:** 10.1093/jisesa/iead045

**Published:** 2023-09-18

**Authors:** Lucas Fonseca de Lacerda, Aloisio Coelho, Pedro Holtz de Paula, Deoclécio J Amorim, Clarice G B Demetrio, José Roberto Postali Parra

**Affiliations:** Department of Entomology and Acarology, “Luiz de Queiroz” College of Agriculture (ESALQ), University of São Paulo (USP), Piracicaba, São Paulo, Brazil; Department of Entomology and Acarology, “Luiz de Queiroz” College of Agriculture (ESALQ), University of São Paulo (USP), Piracicaba, São Paulo, Brazil; Department of Entomology and Acarology, “Luiz de Queiroz” College of Agriculture (ESALQ), University of São Paulo (USP), Piracicaba, São Paulo, Brazil; Department of Exact Sciences, “Luiz de Queiroz” College of Agriculture (ESALQ), University of São Paulo (USP), Piracicaba, São Paulo, Brazil; Department of Exact Sciences, “Luiz de Queiroz” College of Agriculture (ESALQ), University of São Paulo (USP), Piracicaba, São Paulo, Brazil; Department of Entomology and Acarology, “Luiz de Queiroz” College of Agriculture (ESALQ), University of São Paulo (USP), Piracicaba, São Paulo, Brazil

**Keywords:** fall armyworm, quality control, sustainability, egg parasitoid

## Abstract

The widely distributed, polyphagous fall armyworm, *Spodoptera frugiperda* (J.E. Smith, 1797), is one of the most important crop pests worldwide. The egg-parasitoid wasp, *Telenomus remus* Nixon, 1937, is frequently described as a possible control agent for *S. frugiperda*. We selected an isoline of *T. remus* and evaluated its parasitism potential (for 24 h) in *S. frugiperda* eggs, in laboratory conditions, and also its ability to fly at different temperatures and relative humidity levels, aiming to provide basic information about this isoline. The selected isoline maintained good flight capacity without affecting its parasitism efficiency or developing inefficient haplotypes for biological-control programs, compared across generations to a regularline laboratory-reared for more than 60 generations. The flight capacity of the isoline was best at 25–30 °C and relative humidity 70–90%.

The genus *Spodoptera* Guenée includes several species of insect pests, in particular the fall armyworm, *S. frugiperda* (J.E. Smith, 1797), which has 350 species of host plants (Montezano et al. 2018), including important crops such as corn (maize), cotton, soybeans, and rice (Nagoshi 2009, Bueno et al. 2011). Restricted to the Americas until 2016, the species gained more notoriety after it invaded Africa, Asia, the Middle East, and Oceania (Goergen et al. 2016, Ganiger et al. 2018, Jiang et al. 2019, Sharanabasappa et al. 2019) Currently, *S. frugiperda* is reported in more than 70 countries outside the Americas (EPPO 2020). Agrochemicals and genetically modified plants are the main methods presently used to control *S. frugiperda*. However, the results have not been as expected, and for this reason, to meet the concepts of integrated pest management (IPM) and sustainability of modern agriculture, biological control is being increasingly adopted into IPM (Adamczyk Jr and Sumerford 2001, van Lenteren et al. 2018, Bentivenha et al. 2019)

The microhymenopteran *Telenomus remus* (Nixon, 1937) is an egg parasitoid of lepidopterans of the families Pyralidae, Arctiidae, and Noctuidae, with a preference for noctuid hosts, especially members of the genus *Spodoptera* (Cave 2000). *Telenomus remus* is a potential control agent for *S. frugiperda*, due to its high biotic potential and ability to parasitize the egg masses of *Spodoptera* spp., which have eggs in overlapping layers and covered with scales (Ashley 1986, Cave 2000, Molina-Ochoa et al. 2003). *Telenomus remus* is used as a control agent of *Spodoptera* spp. in several countries and regions, including Guyana, Suriname, Colombia, Venezuela, and the Caribbean (Joshi et al. 1982, Cock et al. 1985, Hernández et al. 1989, García-Roa et al. 2002, Singh 2004, Ferrer 2021). However, little information about the methods and evaluation criteria adopted in these studies is available.

Despite these reports, some countries, such as Brazil, that attempt to use *T. remus* as a control agent for *S. frugiperda* have not been successful (Varella et al. 2015). Naranjo-Guevara et al. (2020) demonstrated that maintenance of *T. remus* in laboratory colonies for long periods leads to loss of its flying and foraging capacity, essential for good performance in the field. This loss of quality may be the cause of the divergent results for efficiency of *T. remus* in the field, as reviewed by Colmenarez et al. (2022). Laboratory-rearing conditions can select for haplotypes that will be inefficient in field conditions, since natural enemies are often confined in a spatially limited environment with unlimited supplies of hosts or prey, not giving any adaptative advantages to individuals with good flight capacity (Bartlett 1985). In the case of *T. remus*, the parasitoid was found naturally occurring in Brazil in the year 2021, indicating that it has become established in the Brazilian agro-ecosystem (Wengrat et al. 2021). Based on this discovery, to ensure the maintenance of the “wild” qualities (flight, foraging, and parasitism capacity) of *T. remus* kept in the laboratory, isolines may be an option (Coelho et al. 2016, de Paula et al. 2022). According to Parra and Coelho (2022), the determination of basic biological parameters is highly important in biological-control programs. Therefore, the present study aimed to determine biological parameters of a *T. remus* isoline, comparing its flight capacity and parasitism (over 24 h) with a regular line long maintained in the laboratory, as well as to evaluate the effects of abiotic factors (temperature and relative humidity [RH]) on the flight capacity of the selected isoline, aiming toward its use in an augmentative biological-control program for *S. frugiperda.*

## Materials and Methods

### *Spodoptera frugiperda* Rearing

The laboratory egg masses used in this study were obtained from an established colony of *S. frugiperda* in the Insect Biology Laboratory of the Department of Entomology and Acarology of the University of São Paulo, “Luiz de Queiroz” College of Agriculture (USP/ESALQ). The colony has been maintained for 20 generations since its collection in the field at the Anhumas experimental farm of USP/ESALQ in Piracicaba, São Paulo (22°46ʹ25.1″S, 47°57ʹ49.8″W). Insects were reared on the Greene diet (Greene et al. 1976) with methods described by Parra (2001). The first- and second-instar larvae were pre-inoculated in 200-ml plastic containers containing the artificial diet and kept in a climate-control chamber regulated at 25 ± 1 °C, 70 ± 10% RH, and 14-h photophase, until they reached the pupal stage. The pupae were placed in individual Petri dishes and kept in climate-control chambers under the same conditions until the adults emerged. The adults were transferred in a 1:2 ratio (male:female) to PVC cages (15 cm diameter × 20 cm H) covered inside with sheets of bond paper that served as a laying substrate. The paper sheets were changed daily to collect the eggs.

### *Telenomus remus* Rearing

The *T. remus* parasitoids were obtained from the existing colony at the Insect Biology Laboratory and founded from individuals collected at the Areão ESALQ experimental farm in Piracicaba (22°41ʹ53″ S, 47°38ʹ30″ W). The wasps were reared in *S. frugiperda* egg masses from the laboratory colony. The egg masses were offered to the parasitoids in glass tubes (1.5 cm diameter × 7 cm H) containing droplets of pure honey as food for the adult parasitoids. After 24 h of parasitism, the egg masses were transferred to new glass tubes (1.5 cm diameter × 7 cm H) that also contained droplets of pure honey to feed the adults that emerged. The tubes were kept in climate-controlled chambers set at a temperature of 25 ± 1 °C, RH 70 ± 10%, and 14-h photophase.

### Selection of *T. remus* Isoline

The isoline used was selected from the *T. remus* line collected in the municipality of Piracicaba. Flight-test units, proposed by Dutton and Bigler (1995) were adapted to select flying individuals (de Paula et al. 2022). The adapted flight-test units were composed of PVC cylinders (15 cm diameter × 20 cm H) coated with black paper inside to encourage flight. To select the flying individuals, instead of using a Petri dish with entomological glue, which collect and kill the flying individuals, egg masses of *S. frugiperda* were attached to the inner side of the Petri dishes, in order to attract and select only flying individuals (males and females) from the population. On the referred black paper, a 0.5-cm-wide ring of glue was applied with a glue stick, 3.5 cm from the base of the cylinder, to capture walking insects.

Forty-eight hours after the wasps emerged, the card with the *S. frugiperda* egg mass attached at the top of the cage was collected and the parasitized eggs were used again to repeat the above-mentioned procedure for 3 more generations. After 3 generations, a single female was removed from the population and kept in a tube containing honey droplets and a freshly laid, nonparasitized *S. frugiperda* egg mass. After emergence of the F1 generation, a couple (of siblings) was again selected from the population and transferred to a new glass tube containing a newly laid, nonparasitized *S. frugiperda* egg mass. This procedure (inbreeding) was repeated for 9 successive generations, thus producing a population with a potential heterosis variability of less than 0.14%, and DNA variability lower than 0.01%, considered a pure isoline (Li 1955, Coelho et al. 2016). The selected isoline was maintained for approximately 13 generations under laboratory conditions before the other tests in the study were carried out.

### Biology of *T. remus* Isoline in *S. frugiperda*

The biology of *T. remus* in *S. frugiperda* eggs from a laboratory-rearing colony was studied following the method proposed by Beserra and Parra (2004), where eggs of *S. frugiperda* less than 24 h old were offered daily to newly emerged females of *T. remus*, with approximately 100 ± 15 eggs per female (Bueno et al. 2010). The eggs were offered in glass tubes (1.2 cm diameter × 7.5 cm H) containing a droplet of pure honey as a food source, for a period of 24 h.

For the biology studies, 20 repetitions were carried out. The following parameters were evaluated: duration of development (egg–adult), parasitism survival, daily oviposition rate, total fecundity, number of adults emerged per egg, sex ratio (sr = number of females/number of females + number of males), duration of the embryonic period, and longevity of the parasitoids.

### Flight Capacity and Parasitism Comparison

To determine whether the selected isoline had favorable characteristics for performance in the field, these wasps’ flight capacity and parasitism were compared to a regular line. The regular line has been kept in laboratory conditions since 2010 (Naranjo-Guevara et al. 2020). In 2017, wasps from this line were released in the field and recollected on eggs of *S. frugiperda*, considered as a reintroduction. Since 2017, this regular variable line has been laboratory maintained for 60 generations.

The parasitism capacity of the regular line and isoline of *T. remus* was determined by placing 20 females of regular line and 25 of isoline, newly emerged (24 h older and copulated) and with no prior experience of parasitism, into individual glass tubes containing droplets of pure honey and 100 ± 15 eggs of *S. frugiperda*.

The eggs were exposed to parasitism for 24 h; the tubes were kept at a temperature of 25 ± 1 °C, RH 70 ± 10%, and photophase 14 h until all adults emerged. After emergence, the adults were killed by freezing at −20 °C and counted to determine the number of *T. remus* emerged, since it has already been shown that only one *T. remus* adult can emerge per egg of *S. frugiperda* (Schwartz and Gerling 1974). The egg masses were also checked for nonviable eggs and parasitized eggs that did not produce adult wasps. The parasitism in 24 h of the selected isoline was determined based on the study of its biology and later compared with the parasitism of the regular line.

The flight capacity of each line was evaluated by using flight-test units of the ESALQ model (Prezotti et al. 2002). However, this time, the 15.5-cm Petri dish used to cover the top of the cage contained entomological glue to capture the flying adults of *T. remus* (de Paula et al. 2022).

Flight was allowed for 48 h, after which the flight cages were transferred to a freezer (−20 °C) to kill the adults and facilitate counting of individuals. Adults of *T. remus* collected on the Petri dish at the upper cage were considered to have been flying, those trapped in the glue ring as walkers, and adults at the base of the cylinder and inside the glass tube as nonflyers. Eight replications were performed for each line, and the lines were maintained at 25 ± 1 °C, RH 70 ± 10%, and photophase 14 h.

### Flight Capacity of *T. remus* Isoline at Different Temperatures and RH Levels

The effects of abiotic factors (temperature and RH) on the flight capacity of the *T. remus* isoline were studied separately, using flight-test units of the ESALQ model according to the above-mentioned method. To evaluate the effect of the temperatures 18, 20, 25, 30, and 35 °C, 5 climatic chambers were set at each temperature with ±1 °C of error. In order to not have effect of RH, this abiotic factor was kept constant at 60 ± 5% in each climatic chamber. The assay to evaluate the effect of RH, 30%, 50%, 70%, and 90%, was carried out in another 4 climatic chambers, each one set up in the mentioned RH with ±5% error, and the temperature was kept constant, 25 ± 1 °C. For each treatment, 10 repetitions were prepared, each consisting of one flight-test unit.

### Data Analysis

The parasitism data for the 2 lines were analyzed using the generalized linear model, assuming a Poisson distribution, including quasi-Poisson models when overdispersion was observed (McCullagh and Nelder 1989, Demétrio et al. 2014).

For flight-test data, a multinomial regression model was used. For this simple model, the underlying proportions were assumed to be fixed. However, for the flight-test data in this study, the actual variation was higher than what would be predicted by the multinomial model. In this case, a distribution commonly used to accommodate overdispersion was the Dirichlet multinomial model (Kim et al. 2018).

Using the MGLM package (Zhang and Zhou 2016), the Dirichlet multinomial regression model was fitted to the data for flying, walking, and nonflying individuals, including the line effect. The likelihood ratio (LR) test was used to assess the significance of the effects between the full model and the reduced model. Differences among the proportions of individuals in each class in the different lines were evaluated based on bootstrap confidence intervals with 95% confidence (95% CI), assuming normality. The calculations and the statistical analysis were performed with R 4.2.3 (R Core Team 2018).

## Results

### Biological Parameters of *T. remus* Isoline in *S. frugiperda* Egg Masses

The high survival rate (96%) observed in the study of biological parameters of the *T. remus* isoline indicates that *S. frugiperda* is a suitable host. The other biological parameters determined are total parasitism of 151.5 ± 10.6 eggs, parasitism survival of 96.2 ± 6.2%, sex ratio of 0.6 ± 0.1, egg–adult period of 15.3 ± 2.8, longevity of 14.2 ± 2.7 and 1.0 ± 0.02 *T. remus* emerged per eggs of *S. frugiperda*.

**Fig. 1.**
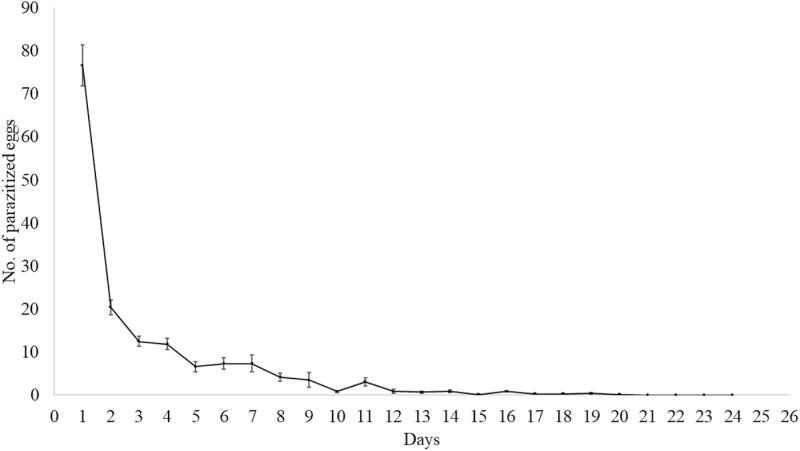
Daily parasitism of *Telenomus remus* isoline on *Spodoptera frugiperda* eggs. Temperature 25 ± 1 °C; RH 70 ± 10%; photophase 14 h; error bars indicate the standard error.

Regarding the parasitism of the *T. remus* isoline, about 50% (mean of 76.5) of the eggs were parasitized in the first 24 h (Fig. 1).

### Flight Capacity and Parasitism Comparison

**Fig. 2.**
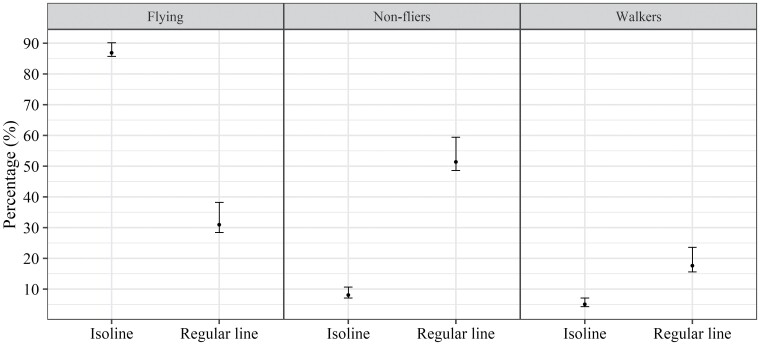
Percentage of flying, nonflying, and walking individuals of *Telenomus remus* isoline compared with a regular line (kept for more than 60 generations in the laboratory). Temperature 25 ± 1 °C; RH 70 ± 10%; photophase 14 h; bars indicate bootstrap 95% CI.

The Dirichlet multinominal model indicated a significant difference between the flight capacity of the 2 *T. remus* lines (LR = 61.45; df = 3; *P* ≤ 0.001); that is, the proportions of flying, walking, and nonflying individuals differed between the lines. For the isoline, flying insects comprised the highest proportion (87%), while for the regular line, kept in the laboratory for more than 60 generations, flying insects comprised only 31%, with a predominance of nonflying insects (51%) (Fig. 2).

The parasitism capacity in 24 h of the regular *T. remus* line did not differ significantly from the capacity of the isoline (*F*_1, 43_ = 0.11, *P* = 0.89). The average parasitism observed in 24 h was 76.5 eggs for the isoline and 81.30 eggs for the regular line.

### Flight Capacity of the *T. remus* Isoline at Different Temperatures and RH Levels

**Fig. 3.**
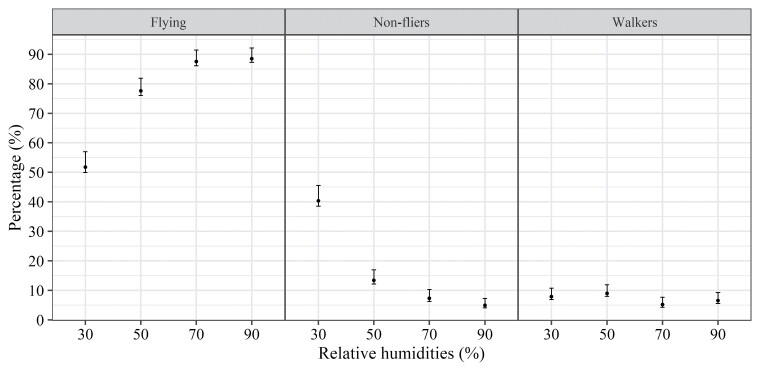
Percentage of flying, nonfliers, and walkers of the *Telenomus remus* isoline emerged from *Spodoptera frugiperda* eggs at 5 different temperatures, RH of 70 ± 5%, and photophase of 14 h; bars indicate bootstrap 95% CI.

Analysis of the data from the flight test of *T. remus* at different temperatures, through the Dirichlet multinomial model, indicated that the temperature significantly affected the flight capacity of the insects (LR = 157.7951; df = 12; *P* < 0.001), with differences in the proportions of flying, walking, and nonflying individuals at the different temperatures. The percentage of flying individuals increased proportionally to the thermal increase, with the highest percentage of flying individuals in the range from 25 to 30 °C. In the 35 °C treatment, the percentage of flying individuals decreased significantly (80%) (Fig. 3).

The response of nonflying individuals was inversely proportional to the increase in temperature, with the lowest percentages of nonflying individuals from 25 to 30 °C, showing that this is the favorable thermal range.

**Fig. 4.**
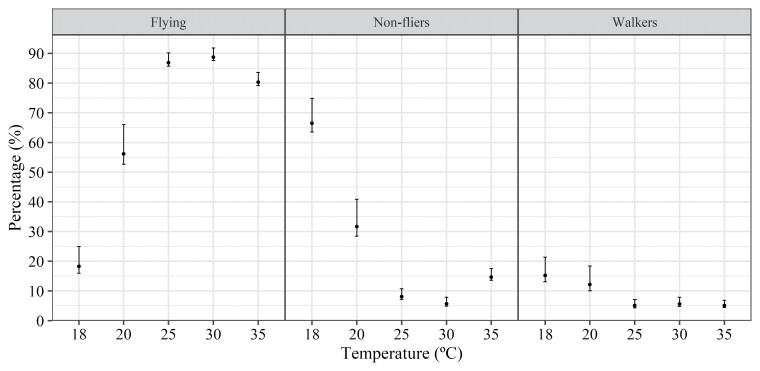
Percentage of flying, nonfliers, and walkers of the *Telenomus remus* isoline emerged from *Spodoptera frugiperda* eggs at 4 different RH levels, temperature of 25 ± 1 °C, and photophase of 14 h; bars indicate bootstrap 95% CI.

The results obtained in the flight test of *T. remus* at different RH levels showed a significant effect (LR = 80.56; df = 9; *P* < 0.001) of RH on the flight capacity of the insects; that is, the proportions of flying, walking, and nonflying individuals differed at the different RH levels. Similarly, to the temperature tests (°C), the proportion of flying individuals increased proportionally to the increase in RH. The highest RH levels, 70 and 90%, were most favorable for the flight activity of the *T. remus* isoline (Fig. 4).

## Discussion

The observed biological parameters of the selected *T. remus* isoline indicated that the selection process did not significantly affect the biology of this parasitoid, since the parameters were in agreement with those described for genetically variable strains of the same species.

Regarding the flight capacity of the selected isoline and the regular line of *T. remus* kept in the laboratory for more than 60 generations, the proportion of flying individuals from the isoline (80%) was significantly higher than the regular line (30%). This indicates that selection of individuals with favorable characteristics and selection of an isoline from these individuals succeeded in maintaining the haplotype responsible for good flight capacity in the population. Similarly, Naranjo-Guevara et al. (2020) evaluated the flight capacity of a “wild” strain of *T. remus* and another strain kept in the laboratory for more than 500 generations and found that the proportion of flying individuals from each strain was 87% and 34%, respectively.

The loss of quality of natural enemies in mass-rearing conditions normally is associated with low genetic diversity leading to increased inbreeding. However, *T. remus* is an arrhenotokous haplodiploid parasitoid, not subject to inbreeding depression because the haploid males, generated by parthenogenesis, express the deleterious alleles and die, “purifying the population” (Henter 2003, West et al. 2005). This indicated that the loss of its ability to forage and fly must be related to the selection pressure of its rearing environment (Luna and Hawkins 2004, Prezotti et al. 2004, Coelho Jr. et al. 2016). Laboratory conditions may impose selection pressure on a population, favoring individuals less adapted to the field and more adapted to laboratory conditions (Coelho et al. 2016), for instance. Mass-reared natural enemies are usually confined in small environments with their hosts or prey, there being no adaptive advantage for individuals with good flight ability (Bartlett 1985).

The loss of quality of *T. remus* caused by the rearing environment may be the factor responsible for some reports of failure in using it as a control agent for *S. frugiperda*. For instance, Varella et al. (2015) reported that a release of approximately 200,000 *T. remus* per hectare in corn fields had no significant effect on the control of *S. frugiperda*. Bueno et al. (2010) studied the same line and recorded a parasitism of 35.7 eggs, lower than the rate found for the isoline in this study (151.5 parasitized eggs). The strain used by Varella et al. (2015) and Bueno et al. (2008) was maintained in the laboratory for more than 20 yr, which may reinforce the hypothesis of selection of less efficient haplotypes for the control of *S. frugiperda* over generations in the laboratory.

Regarding parasitism in 24 h, no significant difference was observed between the genetically variable line and the isoline. This indicates that the selection process of isoline preserved good flight capacity in the population, without an adaptive cost that could affect another characteristic of a biological-control agent.

Therefore, the use of an isoline to mitigate or prevent the loss of quality of natural enemies as a result of their rearing environment can help to ensure quality control in mass rearing of insects for augmentative biological-control programs, once specific trait or traits can be selected (Nunney 2003). Another important factor in quality control is the performance of control agents under different abiotic conditions. Mass-rearing procedures generally provide stable environmental conditions of photoperiod, temperature, and RH, with no selection pressure for individuals capable of overcoming adverse climatic conditions (Lerner 1958).

In the flight tests carried out to evaluate the effect of temperature on the flight capacity of the *T. remus* isoline, the percentage of flying individuals increased proportionally to the temperature increase up to 30 °C, with the best flight capability between 25 and 30 °C. The significant drop in the percentage of flying individuals at 35 °C was probably caused by the proximity to the insect’s upper thermal threshold, which is 35.9 °C (Melo 2022). At 25 °C, the results observed in this study are close to the values found by Naranjo-Guevara et al. (2020) for the same temperature.

Regarding the effect of RH on the flight ability of the selected *T. remus* isoline, the percentage of flying individuals increased with increasing RH. Cave (2000) mentioned that the air RH is one of the most important abiotic factors in rearing *T. remus*. Gupta and Pawar (1985) and Gautam (1986) also demonstrated that higher parasitism of *T. remus* occurs at high RH levels.

Aiming at an augmentative biological control program for *S. frugiperda*, this research demonstrated that an isoline can be a good option for maintaining the quality of *T. remus*. Flight ability was affected by temperature, with the highest levels, 86% and 88%, found in the range of 25–30 °C; temperatures below 25 °C (18 and 20 °C) and above 30 °C (35 °C) negatively affected this parameter. RH also affected the proportion of flying *T. remus*, with the insects flying more at RH levels above 70% (>85% of flying individuals). In conclusion, the selected isoline shows desirable characteristics regarding parasitism and laboratory flight ability; however, for field releases, abiotic factors that may affect the effectiveness of the biological-control agent must be considered.
